# Is the C-reactive protein/albumin ratio a prognostic and predictive factor in sudden hearing loss?^☆^

**DOI:** 10.1016/j.bjorl.2018.10.007

**Published:** 2018-11-22

**Authors:** Ramazan Öçal, Fatma Ceyda Akın Öçal, Mustafa Güllüev, Necat Alataş

**Affiliations:** a Health Sciences University, Ankara Training and Research Hospital, Department of Otolaryngology Head and Neck Surgery, Ankara, Turkey; b Health Sciences University, Gülhane Training and Research Hospital, Department of Otolaryngology Head and Neck Surgery, Ankara, Turkey; c Health Sciences University, Konya Training and Research Hospital, Department of Otolaryngology Head and Neck Surgery, Konya, Turkey

**Keywords:** Sudden hearing loss, C-reactive protein, Albumin, Chronic inflammation, Perda auditiva súbita, Proteína C-reativa, Albumina, Inflamação crônica

## Abstract

**Introduction:**

Sudden hearing loss is a significant otologic emergency. Previous studies have revealed a coexistence of sudden hearing loss with chronic inflammation. The predictive importance of C-reactive protein/albumin values as a prognostic factor has been shown in various inflammatory and tumoral conditions.

**Objectives:**

The aim of this study was to determine whether the C-reactive protein/albumin ratio in sudden hearing loss can be used for prognostic purposes and whether there is a relationship between the neutrophil/lymphocyte ratio and the C-reactive protein/albumin ratio.

**Methods:**

A retrospective examination was made of 40 patients diagnosed with idiopathic sudden hearing loss and a control group of 45 healthy subjects. The pure tone averages of all the patients were determined on first presentation and repeated at 3 months after the treatment. The patients were separated into 2 groups according to the response to treatment. The neutrophil/lynphocyte ratio and the C-reactive protein/albumin ratios were calculated from the laboratory tests.

**Results:**

The patients included 16 females and 24 males with a mean age of 44.1 ± 14.2 years and the control group was composed of 23 females and 22 males with a mean age of 42.2 ± 13.8 years. The mean C-reactive protein/albumin ratio was 0.95 ± 0.47 in the patient group and 0.74 ± 0.13 in the control group. The difference was statistically significant (*p* = 0.009). The mean C-reactive protein/albumin ratio was 0.79 ± 0.12 in the response to treatment group and 1.27 ± 0.72 in the non-response group, with no significant difference determined between the groups (*p* = 0.418). The mean neutrophil/lymphocyte ratio was 3.52 ± 3.00 in the response to treatment group and 4.90 ± 4.60 in the non-response group, with no statistically significant difference determined between the groups (*p* = 0.261).

**Conclusion:**

C-reactive/albumin ratio was significantly higher in patients with sudden hearing loss than in the control group. Although C-reactive protein/albumin ratio was found to be lower in sudden hearing loss patients who responded to treatment compared to those who did not, the difference between two groups was not statistically significant.

## Resumo

**Introdução:**

A perda auditiva neurossensorial súbita ou surdez súbita é uma emergência otológica significativa. Estudos anteriores revelaram uma coexistência dessa condição com inflamação crônica. A importância preditiva dos valores da relação proteína C-reativa/albumina como fator prognóstico tem sido demonstrada em várias condições inflamatórias e tumorais.

**Objetivos:**

O objetivo deste estudo foi determinar se a relação proteína C-reativa/albumina na perda auditiva neurossensorial súbita pode ser usada para fins prognósticos e se existe uma associação entre as relações neutrófilo/linfócito e proteína C-reativa/albumina.

**Método:**

Foram avaliados retrospectivamente 40 pacientes com diagnóstico de perda auditiva neurossensorial súbita idiopática e um grupo controle de 45 indivíduos saudáveis. As médias de tons puros de todos os pacientes foram determinadas na primeira consulta e repetidas 3 meses após o tratamento. Os pacientes foram separados em 2 grupos de acordo com a resposta ao tratamento. As relações neutrófilo/linfócito e proteína C-reativa/albumina foram calculadas a partir de testes laboratoriais.

**Resultados:**

Os pacientes incluíam 16 mulheres e 24 homens, com média de 44,1 ± 14,2 anos, e o grupo controle por 23 mulheres e 22 homens, com média de 42,2 ± 13,8 anos. A média da relação proteína C-reativa/albumina foi de 0,95 ± 0,47 no grupo de pacientes e de 0,74 ± 0,13 no grupo controle e a diferença foi estatisticamente significante (*p* = 0,009). A média da relação proteína C-reativa/albumina foi de 0,79 ± 0,12 do grupo com resposta ao tratamento e de 1,27 ± 0,72 no grupo sem resposta, sem diferença significante entre os grupos (*p* = 0,418). A média da relação neutrófilo/linfócito foi de 3,52 ± 3,00 no grupo com resposta ao tratamento e de 4,90 ± 4,60 no grupo sem resposta, sem diferença estatisticamente significativa entre os grupos (*p* = 0,261).

**Conclusão:**

A relação proteína C-reativa/albumina foi significantemente maior nos pacientes com perda auditiva neurossensorial súbita do que no grupo controle. No entanto, embora a relação proteína C-reativa/albumina tenha sido menor nos pacientes com perda auditiva neurossensorial súbita que responderam ao tratamento em comparação a aqueles que não apresentaram resposta, a diferença entre os dois grupos não foi estatisticamente significante.

## Introduction

Sudden hearing loss (SHL) is a commonly encountered audiological emergency that generally develops unilaterally within 72 h^1^ and is defined as sensorineural hearing loss of at least 30 dB at 3 consecutive frequencies. The etiology has not been fully clarified, but vascular obstruction, viral infections and autoimmunity are thought to be responsible.^2^ Although the pathophysiology is not fully known, there has been recent focus on chronic inflammation.^3^, ^4^ Several protocols are used in treatment, but systemic steroid treatment is accepted as the first therapeutic step.^5^ Previous studies have shown that the neutrophil/lymphocyte ratio (NLR) is an inflammatory marker and could be used to determine the prognosis in SHL.^6^, ^7^

C-reactive protein (CRP) is an acute phase reactant. The CRP level can be used for diagnostic purposes in infection and can also be used for the evaluation of the efficacy of treatment.^8^, ^9^ In the early stages of infection, the CRP value is correlated to the severity of inflammation. Albumin (Alb) is a negative acute-phase protein. Although the Alb value decreases in acute inflammation, essentially it decreases in conditions of chronic inflammation and poor nutrition.^10^, ^11^ The determination of serum Alb values in addition to CRP values could be of prognostic value both in the short and long term in inflammation. The predictive importance of CRP/Alb values as a prognostic factor has been shown in various inflammatory and tumor conditions based on inflammation.^12^, ^13^, ^14^, ^15^

The aim of this study was to determine whether the CRP/Alb level in SHL based on chronic inflammation can be used for prognostic purposes, as well as to show the relationship between NLR and CRP/Alb, which are both inflammatory markers.

## Methods

This retrospective study included 40 patients diagnosed with idiopathic SHL at the otorhinolaryngology clinic of a training and research hospital between March 2016 and February 2017, and an age and gender-matched control group of 45 healthy individuals selected from those attending the polyclinic for routine health screening. The patient group was selected from patients admitted to the hospital for 3 days or less due to SHL. Approval for the study was granted by the Ethics Committee of Selçuk University (decision no. 2017/350). Exclusion criteria were a history of smoking, the presence of active infection, diabetes mellitus, hypertension, chronic kidney disease, chronic liver disease, chronic obstructive pulmonary disease, coronary artery disease, or inflammatory intestinal disease, and trauma, chronic otitis media, tumor, Meniere's disease or otosclerosis as causes of hearing loss.

During diagnosis a detailed ear, nose and throat examination was made of all the patients, including microscopic and otoscopic ear examinations, laboratory tests (full blood count, and biochemistry analysis including CRP and albumin), audiological evaluation and Magnetic Resonance Imaging (MRI). Laboratory examinations were performed at the time of admission to the hospital. Treatment of 1 mg/kg methylprednisolone was administered intravenously to all patients routinely, reducing the dosage by 10 mg/day. Patients who received intratympanic steroids and hyperbaric oxygen therapy were excluded from the study. On first presentation, air conduction measurements at 125–8000 Hz, and bone conduction measurements at 250–4000 Hz were taken by the same audiologist using the same device (AC40, Interacoustic, Denmark) and the pure tone averages (PTA) were determined 0.5, 1, 2, and 4 kHz were used to calculate the PTA.

The same measurements were taken again 3 months after the treatment. The response to treatment was classified according to the Siegel criteria^16^ and 2 groups were formed. Those who were Type 1, 2 and 3 according to Siegel formed the response to treatment group and those who were Type 4, the non-response group (Table 1).

**Table 1:** Siegel criteria. Pure Tone Audiometer (PTA): 500, 1000, 2000 and 4000 Hz arithmetic mean.

Type	Evaluation	Explanation
1	Complete recovery	PTA is 25 dB or lower with treatment
2	Partially recovery	The latest PTA is 25–45 dB and more than 15 dB gain
3	Poor recovery	The latest PTA is 45 dB or higher and more than 15 dB gain
4	No recovery	The latest PTA is 70 dB or higher and less than 15 dB gain

In the analysis of the laboratory tests, the NLR was calculated from the absolute neutrophil and the absolute lymphocyte counts and the CRP/Alb ratio was calculated.

### Statistical analysis

Data were analyzed with SPSS version 23.0 software (IBM Corporation, Armonk, NY, USA). Descriptive statistics were stated as mean ± standard deviation (SD). The comparison of differences in age between the patient and control groups was performed using independent samples *t*-test. The importance of the difference in gender between the two groups was analyzed with the Pearson Chi-Square test.

In the comparisons of CRP/Alb within and between the groups, the Mann–Whitney *U*-test was applied and for the within-group comparison of NLR, the *t*-test was used. Variables were examined at a 95% confidence interval. A value of *p* < 0.05 was accepted as statistically significant.

## Results

The patients included 16 females and 24 males with a mean age of 44.1 ± 14.2 years and the control group was comprised of 23 females and 22 males with a mean age of 42.2 ± 13.8 years. There was no statistically significant difference between the groups in respect of age and gender (Table 1). The mean CRP/Alb ratio was 0.95 ± 0.47 in the patient group and 0.74 ± 0.13 in the control group and the difference between the groups was statistically significant (*p* = 0.009). The mean NLR was determined as 3.96 ± 3.59 in the patient group (Table 2).

**Table 2:** Parameters of patient and control groups (mean ± standard deviation).

	Patient group	Control group	*p*-Values
Age	44.1 ± 14.2	42.2 ± 13.8	0.545
Gender	16 K/24 E	23 K/22 E	0.210
NLR	3.96 ± 3.59	–	–
CRP/Alb	0.95 ± 0.47	0.74 ± 0.13	0.009

Independent samples test, Pearson Chi-Square test, Mann–Whitney *U* test.

Of the 40 patients, 27 were in the response to treatment group and 13 were in the non-response group. The mean CRP/Alb ratio was 0.79 ± 0.12 in the response to treatment group and 1.27 ± 0.72 in the non-response group, with no significant difference determined between the groups (*p* = 0.418). The mean NLR was 3.52 ± 3.00 in the response to treatment group and 4.90 ± 4.60 in the non-response group, with no statistically significant difference determined between the groups (*p* = 0.261) (Table 3).

**Table 3:** Comparison of response and non-response to treatment groups according to NLR and CRP/Alb ratios (mean ± standard deviation).

	Response to treatment group	Non-response to treatment group	*p*-Values
NLR	3.52 ± 3.00	4.90 ± 4.60	0.261
CRP/Alb	0.79 ± 0.12	1.27 ± 0.72	0.418

Mann–Whitney *U*-test, *t*-test.

## Discussion

SHL is a significant ear, nose and throat emergency. Recently, NLR and the CRP/Alb ratio have been shown to have prognostic value as markers of inflammation.^6^, ^7^, ^14^ The aim of this study was to determine the prognostic significance of NLR and the CRP/Alb ratio in SHL, which has been shown to progress with chronic inflammation. However, no statistical relationship was determined.

SHL accounts for 1% of sensorineural hearing losses and has been reported at an incidence of 5–20/100,000.^1^ The most important factor in the recovery of SHL is the early initiation of treatment.^1^, ^2^ A definitive etiological factor cannot generally be established and it is accepted to have formed multifactorially. Inflammation, viral infection and hypoxia are the reasons most frequently held responsible.^4^ Previous studies have revealed a coexistence of SHL with chronic inflammation.^3^, ^4^ A strong relationship between cochlear damage and inflammatory markers has been shown in several studies.^17^, ^18^ The aim of steroid treatment used to treat SHL is to reduce inflammation in the inner ear and to provide benefit from the regulatory role of steroids in protein synthesis.^19^

The CRP level increases significantly during infection and inflammation and this increase develops in correlation with the severity of the infection or inflammation. Albumin is a strong marker in the prognosis of diseases related to infection and inflammation, and a decrease is seen in the acute period.^20^, ^21^ At the same time, Alb catabolism is also correlated with acute infection/inflammation severity.^22^ In the light of this knowledge, it was considered in this study that the CRP/Alb ratio could be of predictive value in the evaluation of response to SHL treatment. The changes in pro-inflammatory cytokines are the underlying mechanism of the prognostic value of the CRP/Alb ratio. For example, the pro-inflammatory cytokine, IL-6, plays an important role in the increase in CRP in inflammation. Furthermore, over-expression of IL-6 is related to low levels of albumin. The CRP/Alb ratio can be calculated easily from the routine blood samples taken from patients admitted with a diagnosis of SHL, and in comparison with other inflammatory cytokines such as IL-6, IL-1A, TNF, etc., does not entail any additional costs.

Previous studies have revealed the prognostic value of the CRP/Alb ratio in various cancers and inflammatory diseases.^12^, ^13^, ^14^, ^15^, ^23^ In nasopharyngeal cancers, the prognostic value of the CRP/Alb ratio has been found to be significant for survival and distant metastasis.^24^ The CRP/Alb ratio has also been reported to be a new and promising biomarker to show activity in Crohn's disease.^15^ In the current study, the CRP/Alb ratio was found to be significantly higher in the SHL patients compared to the control group (*p* < 0.05). This also demonstrated inflammation in SHL. However, although the CRP/Alb ratio was found to be lower in the SHL patients who responded to treatment compared to those who did not respond, the difference between the groups was not observed to be statistically significant. This result could suggest that the inflammation in the non-response group was more severe and this could be a guide on the subject of prognosis and recovery. Studies have shown that the NLR is significantly low in groups responding to treatment for SHL and it has been reported that NLR is a rapid, safe indicator in the prediction of prognosis in SHL.^6^ In the current study, the NLR was observed to be lower in the group that responded to treatment, but no statistically significant difference was determined between the groups.

Limitations of this study can be considered to be the low number of subjects and that there was no standardized cutoff value for CRP/Alb and NLR.

## Conclusion

In conclusion, the CRP/Alb ratio shows the presence of inflammation in SHL and may be an indicator for prognosis. This is the first study to have demonstrated that the CRP/Alb ratio shows inflammation in SHL and could be used as a prognostic value.

In conclusion, CRP/Alb ratio was significantly higher in patients with SHL than in control group.

However, although CRP/Alb ratio was found to be lower in SHL patients who responded to treatment compared to those who did not, the difference between two groups was not statistically significant.

This is the first study showing the relationship between SHL and CRP/Alb ratio.

CRP/Alb ratio can be used to determine the severity of inflammation in SHL since it is a inexpensive examination and can be calculated easily.

Furthermore, prospective, controlled, multicentric studies with large populations are needed to determine whether this ratio can be used for SHL prognosis.

## Ethical approval

All procedures performed in studies involving human participants were in accordance with the ethical standards of the institutional and/or national research committee and with the 1964 Helsinki declaration and its later amendments or comparable ethical standards.

## Conflicts of interest

The authors declare no conflicts of interest.
